# Evolution of Cortical Lesions and Function-Specific Cognitive Decline in People With Multiple Sclerosis

**DOI:** 10.1212/WNL.0000000000213650

**Published:** 2025-05-12

**Authors:** Eva A. Krijnen, Julia Jelgerhuis, Maureen Van Dam, Piet M. Bouman, Frederik Barkhof, Eric C. Klawiter, Hanneke E. Hulst, Eva M.M. Strijbis, Menno M. Schoonheim

**Affiliations:** 1 MS Center Amsterdam, Anatomy and Neurosciences, Vrije Universiteit Amsterdam, Amsterdam Neuroscience, Amsterdam UMC location VUmc, the Netherlands;; 2 Department of Neurology, Massachusetts General Hospital, Harvard Medical School, Boston, MA;; 3 MS Center Amsterdam, Radiology and Nuclear Medicine, Vrije Universiteit Amsterdam, Amsterdam Neuroscience, Amsterdam UMC location VUmc, the Netherlands;; 4 Queen Square Institute of Neurology and Centre for Medical Image Computing, University College London, United Kingdom;; 5 Institute of Psychology, Health, Medical and Neuropsychology Unit, Leiden University, the Netherlands;; 6 Leiden Institute for Brain and Cognition, Leiden University, the Netherlands; and; 7 MS Center Amsterdam, Neurology, Vrije Universiteit Amsterdam, Amsterdam Neuroscience, Amsterdam UMC location VUmc, the Netherlands.

## Abstract

**Background and Objectives:**

Cortical lesions in multiple sclerosis (MS) severely affect cognition, but their longitudinal evolution and impact on specific cognitive functions remain understudied. This study investigates the evolution of function-specific cognitive functioning over 10 years in people with MS and assesses the influence of cortical lesion load and formation on these trajectories.

**Methods:**

In this prospectively collected study, people with MS underwent 3T MRI (T1 and fluid-attenuated inversion recovery) at 3 study visits between 2008 and 2022. Cognitive functioning was evaluated based on neuropsychological assessment reflecting 7 cognitive functions: attention; executive functioning (EF); information processing speed (IPS); verbal fluency; and verbal, visuospatial, and working memory. Cortical lesions were manually identified on artificial intelligence–generated double-inversion recovery images. Linear mixed models were constructed to assess the temporal evolution between cortical lesion load and function-specific cognitive decline. In addition, analyses were stratified by MS disease stage: early and late relapsing-remitting MS (cutoff disease duration at 15 years) and progressive MS.

**Results:**

The study included 223 people with MS (mean age, 47.8 ± 11.1 years; 153 women) and 62 healthy controls. All completed 5-year follow-up, and 37 healthy controls and 94 with MS completed 10-year follow-up. At baseline, people with MS exhibited worse functioning of IPS and working memory. Over 10 years, cognitive decline was most severe in attention, verbal memory, and EF. At baseline, people with MS had a median cortical lesion count of 7 (range 0–73), which was related to subsequent decline in attention (B[95% CI] = −0.22 [−0.40 to −0.03]) and verbal fluency (B[95% CI] = −0.23[−0.37 to −0.09]). Over time, cortical lesions increased by a median count of 4 (range −2 to 71), particularly in late and progressive disease, and was related to decline in verbal fluency (B [95% CI] = −0.33 [−0.51 to −0.15]). The associations between (change in) cortical lesion load and cognitive decline were not modified by MS disease stage.

**Discussion:**

Cognition worsened over 10 years, particularly affecting attention, verbal memory, and EF, while preexisting impairments were worst in other functions such as IPS. Worse baseline cognitive functioning was related to baseline cortical lesions, whereas baseline cortical lesions and cortical lesion formation were related to cognitive decline in functions less affected at baseline. Accumulating cortical damage leads to spreading of cognitive impairments toward additional functions.

## Introduction

Multiple sclerosis (MS) is a chronic neuroinflammatory and neurodegenerative disease of the CNS. Although traditionally considered primarily a white matter (WM) disease, gray matter (GM) pathology in MS is common and can be extensive in the form of cortical lesions, with an impact on cognitive impairment at least twice as strong compared with WM lesions.^1,2^ Cognitive impairment in MS is frequently present, affecting up to 65% of individuals,^1^ and substantially affects daily functioning and quality of life.^1,3^ Early impairment is commonly characterized by impaired information processing speed (IPS) and memory^1^ but can progress heterogeneously with different degrees of severity involving one or multiple cognitive functions.^4,5^ Despite the recognized importance of cortical lesions, the lack of longitudinal data remains a key factor contributing to the current lack of knowledge on how cortical lesions evolve over time and how these relate to a decline in specific cognitive functions.

In fact, the few studies describing longitudinal evolution of cortical lesions mostly focus on overall disability progression^2,6^ or general cognitive status^2,7,8^ while studying individual functions could help stratify individuals at risk of different forms of progression. For example, visuospatial memory functioning has been identified as a key function in distinguishing cognitive profiles in MS.^5^ The relationship between cortical lesions and cognitive decline is not straightforward. Clearly, cortical lesion formation is more marked in more progressive disease,^2,6,8,9^ although it has been suggested that cortical lesions formed early in the disease exert greater clinical impact compared with those formed later on.^10^ In this case, early disease shifts from no to some damage may affect cognition more profoundly than incremental damage with substantial cortical damage later. How decline in specific cognitive functions associates with cortical lesion formation across disease stages in MS remains unknown.

The aim of this study was, therefore, to investigate the evolution of function-specific cognitive functioning over 10 years in people with MS and assess the influence of cortical lesion load and formation on these trajectories. In addition, we evaluated whether cortical lesion load, cognitive decline, and their relationship vary across disease stages. By focusing on predefined cognitive functions, this research will contribute to a better understanding of the relationship between cortical lesions and cognitive decline, crucial for developing targeted interventions to mitigate cognitive decline and tailoring monitoring strategies in MS.

## Methods

### Standard Protocol Approvals, Registrations, and Patient Consents

This longitudinal study involves a secondary analysis of the prospectively collected Amsterdam MS cohort, which has been approved by the institutional ethics review board of the Amsterdam UMC (2020.269). All participants provided written informed consent before participation.

### Participants

Included participants were part of the Amsterdam MS cohort.^11,12^ This cohort is derived from 3 distinct subcohorts: a general heterogeneous MS cohort, a cohort with short disease duration, and a longstanding MS cohort. Inclusion criteria included diagnosis of clinically definite MS,^13^ age of 18 years or older, and, for the longstanding cohort specifically, minimum disease duration of 10 years. Healthy controls were recruited through social media and through relatives/contacts of people with MS and hospital staff. Longitudinal data at baseline and 5-year follow-up from this cohort have been used in previous publications,^11,12,14^ but the longitudinal evolution of cortical lesions over 10 years has not been investigated before. Data were collected at up to 3 study visits between 2008 and 2022. Participants with MS had no relapses or steroid treatment for at least 2 months before each study visit. Of the entire cohort, all people with MS and healthy controls (HCs) with MRI and neuropsychological assessment data available at baseline and first (5-year) follow-up were included in this study. Exclusion criteria for all participants were the presence of other neurologic or neuropsychiatric disorders and contraindications to MRI.

For subgroup analysis across MS disease stages, individuals with MS were categorized based on their MS disease type into early and late relapsing-remitting MS (RRMS) and progressive MS (PMS, including both primary PMS and secondary PMS). The distinction between early and late RRMS was based on a disease duration of 15 years at baseline. Because most individuals with secondary progressive MS transition from RRMS to the progressive phase after 10–20 years of disease duration, we selected 15 years as the cutoff to distinguish between “early” and “late” relapsing-remitting disease,^15^ as also reported in earlier work.^9^

### Clinical and Cognitive Assessments

Clinical assessments were performed at all study visits directly before MRI. The Expanded Disability Status Scale (EDSS)^16^ was used by an experienced physician blinded to the imaging results. The Checklist Individual Strength-20^17^ and Hospital Anxiety and Depression Scale^18^ were used to assess fatigue and depression, respectively. To evaluate cognitive functioning, all participants underwent the Brief Repeatable Battery of Neuropsychological Tests,^19^ as well as the Concept Shifting Test, Stroop Color-Word Test, and Memory Comparison Test. Cognitive (sub)test scores can be interpreted in multiple ways and reflect different cognitive functions. For this study, we opted to have (sub)scores of single cognitive tests reflect one specific cognitive function, in line with previous work on this cohort.^11,20^ Hence, (sub)scores of tests were classified into 7 predefined cognitive domains: attention (reflected by the Stroop Color-Word Test^21^), executive functioning (Concept Shifting Test^22^), IPS (Symbol Digit Modalities Test^23^), verbal fluency (Word List Generation Test^19^), verbal memory (Selective Reminding Test^24^), visuospatial memory (Spatial Recall Test^19^), and working memory (Memory Comparison Test^25^). Raw test scores of all participants of each cognitive domain were corrected for effects of age, sex, and education, as previously described.^11^ Test scores were transformed into *Z*-scores based on data of included HCs at the respective time point. To compare the importance of examining individual cognitive functions with overall cognitive functioning, “average cognition” *Z*-scores were calculated by averaging *Z*-scores of individual 7 cognitive functions. Cognitive impairment per function was defined as a *Z*-score less than −1.5 SD relative to HCs, as previously described.^11,26^

#### Change Over Time

Cognitive change during follow-up was defined as the difference in *Z*-scores relative to HCs between time intervals divided by time interval, yielding an “annualized rate of change.” Annualized rates of change were computed for average cognition and each predefined individual function. Separate rates were calculated for baseline to 10-year follow-up and for the 5-year time intervals separately, that is, baseline to 5-year follow-up and 5-year to 10-year follow-up.

### Image Acquisition

All participants underwent 3T MRI (GE, Milwaukee, WI), which received a gradient upgrade between baseline (Signa HDxt) and the 5-year study visit (Discovery MR750). At all study visits, the same 8-channel phased-array head coil was used. The scanning protocol included a 3D T1-weighted fast spoiled gradient-echo sequence (repetition time/echo time/inversion time 7.8/3.0/450 ms, flip angle 12°, 0.94 × 0.94 × 1.00 mm^3^ voxel size) and 3D T2-weighted fluid-attenuated inversion recovery (FLAIR) sequence (repetition time/echo time/inversion time 8,000/125/2,350 ms, 0.98 × 0.98 × 1.20 mm^3^ voxel size). Owing to potential effects of the gradient upgrade between measurements^27^ and the lack of cortical lesion volume data in healthy brains to account for these effects, we included baseline cortical lesion volumes while focusing on cortical lesion counts only longitudinally.

### Data Processing

FLAIR images were used to automatically segment WM lesions through the machine learning method nicMS.^28^ After manual correction, WM lesion volumes were calculated. Lesion Segmentation Toolbox for SPM^29^ was used to fill T1-weighted images for maximizing T1 processing steps.

Cortical surface reconstruction was performed through the longitudinal pipeline of FreeSurfer (v7.0) with filled T1-weighted images of all available time points as input. Segmentations were visually checked, manually corrected by an experienced researcher (E.A.K.), and reran if errors occurred. For descriptive purposes, brain volumes were extracted and corrected for intracranial volume provided by FreeSurfer, yielding normalized brain volumes.

For cortical lesion identification, artificially generated double-inversion recovery (aiDIR) images were generated using in-house software, as previously described.^30^ This process fuses information from T1-weighted and FLAIR sequences to generate aiDIR images. At each time point, cortical lesions were segmented on aiDIR images with Slicer (v5.2.1) by experienced readers (E.A.K. and P.M.B.), in consensus, according to the MAGNIMS guidelines.^31^ Cortical lesion counts were calculated. To limit segmentation bias from confluent WM lesions located in deep WM in cortical lesion volume calculations,^32^ cortical lesion masks were masked by the cortical GM segmentation dilated by 4 mm into subcortical WM before volume calculations.^32^ For that, cortical lesion masks were rigidly transformed to the T1-weighted FreeSurfer image with linear interpolation.

### Statistical Analysis

Details regarding the statistical analysis are reported in the eMethods. In brief, R (v4.2.1) was used for statistical analyses. Highly right-skewed data, that is, lesion count and volume, were log-transformed for subsequent analysis.

#### Baseline Group Comparisons

We analyzed baseline characteristics with descriptive statistics, expressed as N (%) for categorical variables, mean (SD) for continuous normally distributed variables, or median [interquartile range, IQR] for ordinal or continuous non-normally distributed variables. Group comparisons between patients with MS and HCs were conducted using the Student *t* tests for normally distributed continuous data, Mann-Whitney *U* tests for non-normal data, and chi-square tests for categorical data.

#### Temporal Changes in Cognition

Likelihood ratio tests were performed for average cognition, comparing a null model with only random intercepts for participants with a model including a time variable as fixed effect. Given potential bias due to variable follow-up time intervals between participants, a third linear mixed model including follow-up time intervals was tested against the time-only model. This was repeated for each cognitive function separately.

#### Temporal Changes in Cortical Lesions

We performed a likelihood ratio test for log-transformed cortical lesion counts, comparing the null model with only random intercepts for participants with the model including time as fixed effect to assess significant change in cortical lesion counts over time.

#### Cognitive Decline Explained by Cortical Lesion Characteristics

##### Baseline Cognition Explained by Baseline Cortical Lesion Count and Volume

Multivariable linear regression models were constructed, explaining cognition by either baseline cortical lesion count or volume. Age and sex were included as covariates. As an additional step, we incorporated baseline WM lesion volume as covariate in the models to evaluate the specific impact of cortical damage compared with WM damage on the various cognitive functions assessed.

##### Change in Cognition Explained by Baseline Cortical Lesion Count and Volume

As part of our primary aim, we assessed whether baseline cortical lesion count or volume explains any variance in cognitive functioning during follow-up. An extended linear mixed model including baseline cortical lesion count/volume was compared with the time-only model. Again, baseline WM lesion volume was added to assess whether cortical vs WM damage affects cognitive functions differently, compared with baseline cortical lesion models.

##### Change in Cognition Explained by Temporal Change in Cortical Lesion Count

Likewise, a separate linear mixed model was fitted, including the change in cortical lesion count during 10-year follow-up as a fixed factor. This approach was performed for average cognition and each cognitive function separately. For significant associations, we evaluated whether associations were driven by cortical lesion formation or baseline lesion count by comparing models with baseline count added with those using only 10-year changes.

#### Relevance of Disease Stage

For significant associations, we tested whether MS disease stage (i.e., early and late RRMS and PMS) acted as a moderator in the relationships. Models were, therefore, extended to include the interaction term between cortical lesions and MS disease stage.

Effect sizes and beta-coefficients with corresponding 95% CIs were reported. If applicable, *p* values of statistical models were Bonferroni-corrected for multiple comparisons among the 7 cognitive functions, per analysis step, with α-level of 0.05, reported in the main text as *p*_*corr*_.

### Data Availability

Tabular data supporting our findings are available from the corresponding author, on reasonable request.

## Results

### Demographics

This study included 223 people with MS (mean age, 47.8 ± 11.1 years; 153 [68.6%] women), who were similar in age, sex, and education to the 62 included HCs (46.2 ± 9.7 years; 34 [54.8%] women; Table 1). People with MS had predominantly relapsing-remitting disease (77.6%) with a mean disease duration of 17 ± 9 years; were mildly disabled clinically, reflected by a median EDSS score of 3 [2–4]; and experienced more fatigue compared with HCs. People with MS had significant WM and deep GM volume loss compared with HCs, whereas their cortical volumes seemed similar.

**Table 1:** Characteristics of People With Multiple Sclerosis and Healthy Volunteers

Baseline characteristics	Completed 5-y follow-up		Completed 10-y follow-up		HC vs MS (5-y)	HC vs MS (10-y)
	HC	MS	HC	MS		
	N = 62	N = 223	N = 37	N = 94	*p* Value	*p* Value
Time between baseline and 5-y follow-up, years	5.5 ± 1.1	4.8 ± 0.9	5.6 ± 1.1	4.8 ± 0.8	<0.001	<0.001
Time between 5-y and 10-y follow-up, years	—	—	5.8 ± 0.4	6.0 ± 0.8	—	0.086
Demographics						
Age at baseline, y	46.2 ± 9.7	47.8 ± 11.1	46.1 ± 9.4	48.2 ± 10.2	0.279	0.257
Sex (female)	34 (54.8%)	153 (68.6%)	22 (59.5%)	67 (71.3%)	0.062	0.273
Level of education, y	15.1 ± 3.1	14.3 ± 3.2	14.6 ± 3.2	14.3 ± 3.1	0.092	0.665
Disease characteristics						
MS subtype (RR/SP/PP)	—	173 (77.6%)/32 (14.4%)/17 (7.6%)	—	79 (84.0%)/10 (10.6%)/5 (5.3%)	—	—
EDSS score	—	3 [2 – 4]	—	3 [2–4]	—	—
Disease duration, years	—	17.3 ± 9.1	—	17.5 ± 7.8	—	—
CIS20 score	41.2 ± 21.5	72.8 ± 29.3	39.5 ± 17.9	66.4 ± 25.2	<0.001	<0.001
HADS Depression scale score	3.1 ± 12.8	9.4 ± 42.3	1.2 ± 1.7	9.4 ± 42.8	0.056	0.068
Medication at baseline	—	147 (65.9%)	—	60 (63.8%)	—	—
Medication at 5-y follow-up						
Stable treatment	—	168 (75.3%)	—	71 (75.5%)	—	—
Switch in treatment	—	55 (24.7%)	—	23 (24.5%)	—	—
Medication at 10-y follow-up						
Stable treatment	—	—	—	83 (88.3%)	—	—
Switch in treatment	—	—	—	11 (11.7%)	—	—
Clinical relapse between baseline and 5-y follow-up	—	73 (32.7%)	—	28 (29.8%)	—	—
Clinical relapse between 5-y and 10-y follow-up	—	—	—	11 (11.7%)	—	—
MRI						
White matter lesion volume, mL	—	12.8 [6.6–25.1]	—	11.6 [6.5–20.1]		
Cortical lesion volume, mL	—		—			
Cortical lesion count	—		—			
Norm. white matter volume	0.29 ± 0.02	0.28 ± 0.02	0.29 ± 0.02	0.28 ± 0.02	<0.001	0.001
Norm. deep gray matter volume	3.87⋅10^−2^ ± 2.05⋅10^−3^	3.52⋅10^−2^ ± 3.64⋅10^−3^	3.86⋅10^−2^ ± 2.05⋅10^−3^	3.59⋅10^−2^ ± 3.18⋅10^−3^	<0.001	<0.001
Norm. cortical volume	0.33 ± 0.01	0.32 ± 0.02	0.33 ± 0.01	0.33 ± 0.02	0.048	0.570
Cognitive functioning, *Z*-score						
Average cognition	0.06 ± 0.49	−0.64 ± 1.08	0.13 ± 0.5	−0.51 ± 0.76	<0.001	<0.001
Attention	0.02 ± 0.69	−0.51 ± 0.96	0.02 ± 0.75	−0.38 ± 0.79	<0.001	0.009
Executive functioning	−0.02 ± 0.75	−0.88 ± 1.57	0.12 ± 0.68	−0.67 ± 1.55	<0.001	<0.001
Information processing speed	0.12 ± 1.01	−1.00 ± 1.26	0.27 ± 1.00	−0.85 ± 1.27	<0.001	<0.001
Verbal fluency	0.12 ± 1.03	−0.37 ± 1.17	0.18 ± 1.03	−0.30 ± 0.85	0.002	0.015
Verbal memory	0.06 ± 0.91	−0.60 ± 1.18	0.06 ± 0.90	−0.33 ± 1.04	<0.001	0.038
Visuospatial memory	0.10 ± 0.89	−0.38 ± 1.20	0.12 ± 0.89	−0.16 ± 1.10	0.001	0.130
Working memory	0.00 ± 0.80	−1.07 ± 1.57	0.14 ± 0.79	−0.86 ± 1.22	<0.001	<0.001

Abbreviations: CIS20 = Checklist Individual Strength-20; EDSS = Expanded Disability Status Scale; HADS = Hospital Anxiety and Depression Scale; PP = primary progressive; RR = relapsing-remitting; SP = secondary progressive.

Characteristics of included people with multiple sclerosis (MS) and healthy controls (HC) who completed 5-y follow-up and 10-y follow-up. Variables are reported as mean ± SD, median [interquartile range], or number (percentage). Brain volumes were normalized by total intracranial volume. Group comparisons between MS cohorts and HCs were conducted using Student t tests for normally distributed continuous data, Mann-Whitney U tests for non-normally distributed continuous or ordinal data, and chi-square tests for categorical data.

Of the initial cohort that completed 5-year follow-up, 94 people with MS (40.3%) and 37 HCs (59.7%) completed 10-year follow-up (Table 1). One-third of people with MS had a relapse by 5 years, with 24.7% changing therapy, and 11.7% relapsed and switched therapy by 10 years. The 37 HCs who finished 10-year follow-up showed no demographic differences compared with the 25 HCs who completed only 5 years (eTable 1). In the MS group, we observed higher GM volume, lower WM lesion volumes, and better functioning of verbal and visuospatial memory at baseline in the 94 people who completed 10-year follow-up compared with the 129 with only 5-year follow-up data.

### Cross-Sectional Cognitive Performance

At baseline, mean *Z*-scores were lowest for IPS (−1.00 ± 1.26) and working memory (−1.07 ± 1.57), with 90 people with MS (40.4%) showing impairment in at least 2 functions, as previously published.^11,26^ Mean *Z*-scores were least affected in verbal fluency (−0.37 ± 1.17) and visuospatial memory (−0.38 ± 1.20).

**Figure 1:**
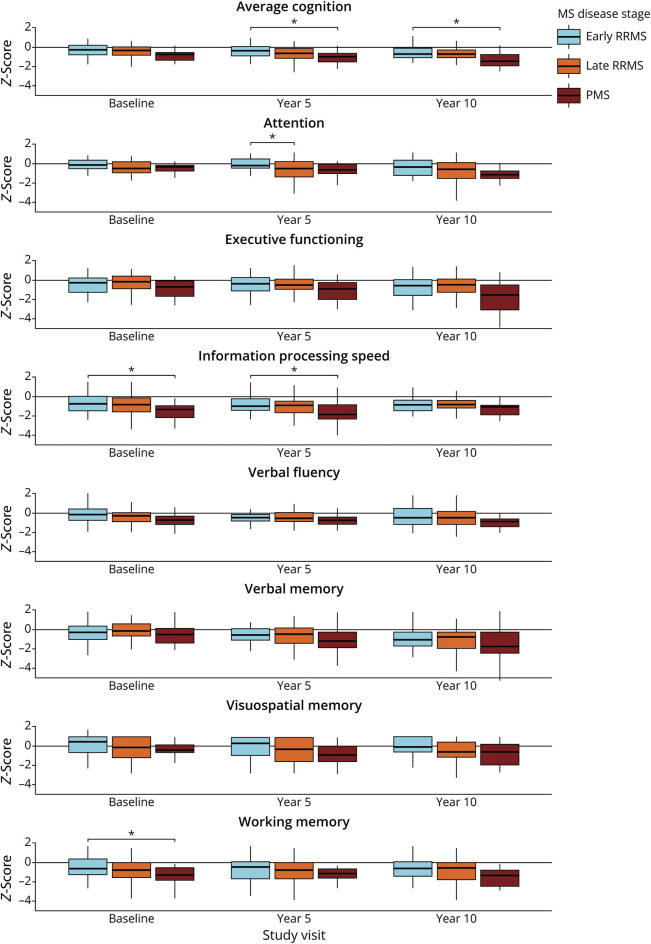
Cognitive Functioning Across Multiple Sclerosis Disease Stages Boxplots of the functioning across cognitive functions, as *Z*-score relative to the included healthy controls, are shown for each study visit, stratified by multiple sclerosis (MS) disease stage: early relapsing-remitting (RR), late RRMS, and progressive (P) MS. Significant differences between disease stages (Bonferroni-corrected *p* value <0.05) are indicated by asterisks.

In the 10-year follow-up group, average cognitive functioning was significantly worse in PMS compared with early RRMS at 5-year (*p*_*corr*_ = 0.048) and 10-year (*p*_*corr*_ = 0.020) follow-up (Figure 1). PMS exhibited poorer IPS functioning compared with early RRMS at baseline (*p*_*corr*_ = 0.015) and at year 5 (*p*_*corr*_ = 0.029), but not at year 10 (*p*_*corr*_ = 0.276). Working memory at baseline was significantly worse in PMS compared with early RRMS (*p*_*corr*_ = 0.044). Attention functioning at year 5 was worse in late RRMS compared with early RRMS (*p*_*corr*_ = 0.017). Other functions were similar across disease stages.

### Temporal Change in Cognition

**Figure 2:**
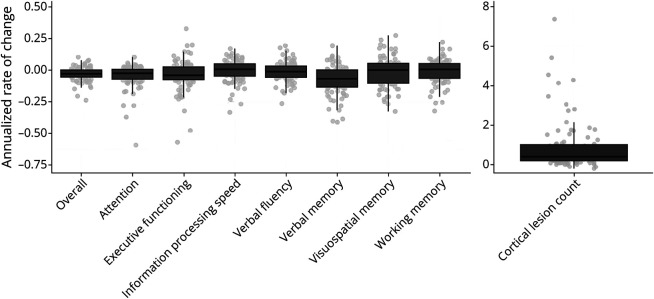
Annualized Rate of Change in Cognitive Functioning and Cortical Lesion Count Over 10 Years Boxplots of the annualized rates of change are shown for average cognition (referred to as “overall”) and the 7 cognitive functions (left panel) and for the cortical lesion count (right panel).

Over 10 years, people with MS exhibited an annualized decrease in average cognitive functioning (annualized, mean −0.03 ± 0.05 per year), particularly of attention (−0.05 ± 0.09) and verbal memory (−0.08 ± 0.11; Table 2 and Figure 2), predominantly during the first 5-year period (eTable 2). Annualized decreases in attention and verbal memory were evident in all disease stages, but most pronounced in late RRMS and PMS. Remarkably, annualized decreases in IPS were only evident in early RRMS (−0.03 ± 0.09 vs −0.01 ± 0.08 in late RRMS and 0.03 ± 0.07 in PMS).

**Table 2:** Annualized Rate of Change in Cognitive Functioning Over 10-Year Follow-Up

Annualized change	HC	MS	Early RRMS	Late RRMS	PMS
	N = 37	N = 94	N = 34	N = 45	N = 15
Average cognition	−0.01 ± 0.03	−0.03 ± 0.05	−0.02 ± 0.03	−0.03 ± 0.07	−0.04 ± 0.05
Attention	0.00 ± 0.04	−0.05 ± 0.09	−0.03 ± 0.05	−0.05 ± 0.12	−0.07 ± 0.08
Executive functioning	−0.01 ± 0.06	−0.04 ± 0.12	−0.02 ± 0.07	−0.04 ± 0.14	−0.08 ± 0.16
Information processing speed	−0.02 ± 0.08	−0.01 ± 0.09	−0.03 ± 0.09	−0.01 ± 0.08	0.03 ± 0.07
Verbal fluency	−0.01 ± 0.08	−0.01 ± 0.08	−0.02 ± 0.06	−0.01 ± 0.09	−0.02 ± 0.08
Verbal memory	−0.01 ± 0.07	−0.08 ± 0.11	−0.05 ± 0.09	−0.09 ± 0.11	−0.10 ± 0.16
Visuospatial memory	0.00 ± 0.12	−0.03 ± 0.12	0.00 ± 0.11	−0.04 ± 0.12	−0.06 ± 0.10
Working memory	−0.01 ± 0.06	−0.01 ± 0.09	−0.02 ± 0.07	0.00 ± 0.10	−0.02 ± 0.10

Abbreviations: PMS = progressive MS; RRMS = relapsing-remitting MS.

Annualized rates of change in cognition over 10-y follow-up is defined as the difference in cognition *Z*-scores relative to the included healthy controls (HC) divided by the time interval and reported as mean ± SD. Annualized rates of change are displayed for the entire multiple sclerosis (MS) sample and for each MS disease stage separately.

Then, we tested significant change over time in cognitive functioning through linear mixed regression models. Average cognition functioning showed significant decline over 10-year follow-up (χ^2^ [2] = 41.9, *p* < 0.001). At year 5, the estimate of the effect indicated a negative change compared with baseline (B [95% CI] = −0.20 [−0.30 to −0.11], *t* = −4.13). At year 10, the change of the effect was greater than at year 5 (B [95% CI] = −0.3 [−0.43 to −0.24], *t* = −6.76). However, relatively, the largest effect was evident in the first 5-year follow-up. The inclusion of follow-up time intervals in the model did not significantly improve its fit to the data (χ^2^ [2] = 3.3, *p* = 0.195). As a result, follow-up time intervals were excluded from further models.

Similar findings were observed for attention (χ^2^ [2] = 26.0, *p*_*corr*_ < 0.001), EF (χ^2^ [2] = 11.0, *p*_*corr*_ = 0.029), and verbal memory (χ^2^ [2] = 42.9, *p*_*corr*_ < 0.001). IPS (χ^2^ [2] = 3.0, *p*_*corr*_ > 0.999), verbal fluency (χ^2^ [2] = 6.1, *p*_*corr*_ = 0.335), and visuospatial (χ^2^ [2] = 6.7, *p*_*corr*_ = 0.246) and working (χ^2^ [2] = 1.6, *p*_*corr*_ > 0.999) memory did not show significant change over time (eTable 3 shows detailed statistics).

### Temporal Change in Cortical Lesions

Of 223 people with MS, 199 (89.2%) had cortical lesions at baseline. Cortical lesion counts tended to be higher in patients with PMS (median 14.0 [IQR 4.5–26]) compared with patients with early RRMS (4.5 [2.0–9.0], *p*_*corr*_ = 0.051). New cortical lesions formed in 139 people (62.3%) during 5 years. Cortical lesion counts remained stable in 63 people (28.4%), and 21 (9.5%) showed a decrease in cortical lesions over 5 years. Of 94 individuals who completed 10-year follow-up, new cortical lesions formed in 81 people (86.2%) during the second 5-year follow-up while cortical lesion count remained stable in 10 (10.6%) and decreased in only 3 people with MS (3.2%).

Individuals [n = 86 (94.5%)] showing increases in cortical lesion counts during the entire 10-year follow-up exhibited higher cortical lesion counts and volumes at baseline compared with ones with stable (n = 5 [5.3%]) or decreasing (n = 3 [3.2%]) cortical lesion counts over time (eTable 4). They also showed lower WM volume while cortical and deep GM volumes were similar to the individuals with stable or decreasing cortical lesion counts over time.

Cortical lesions significantly increased with a median count of 4 [1.9–10.1] over the entire 10-year period. Figure 2 shows the annualized increase in the cortical lesion count over 10-year follow-up. Increases in log-transformed cortical lesion counts were significant over time (χ^2^ [2] = 165.9, *p* < 0.001). At year 5, cortical lesion counts were higher compared with baseline, reflected by a positive change of the estimate (B [95% CI] = 0.13 [0.06; 0.19], *t* = 3.9). At year 10, the change was greater than at year 5 (B [95% CI] = 0.52 [0.45–0.58], *t* = 15.6). Relatively, the largest effect was evident in the second 5-year follow-up. The inclusion of follow-up time intervals in the model did not significantly improve its fit to the data (χ^2^ [2] = 0.48, *p* = 0.7867). When controlling for MS disease stage, log-transformed cortical lesion counts still showed significant increases over 10 years (χ^2^ [2] = 6.9, *p* = 0.031), in which late RRMS (B [95% CI] = 0.46 [0.01–0.92], *t* = 1.97) and PMS (B [95% CI] = 0.77 [0.15–1.40], *t* = 2.42) showed greater cortical lesion count increases compared with early RRMS.

### Cognitive Decline Explained by Cortical Lesion Characteristics

#### Baseline Cognition Explained by Baseline Cortical Lesion Count and Volume

Baseline cortical lesion counts and volumes were associated with baseline cognitive functioning, specifically attention, EF, IPS, and verbal and working memory. When considering WM lesion volume, cortical lesion counts and volumes were still associated with baseline cognitive functioning, particularly attention, EF, and working memory. IPS seemed significantly related to WM lesion volume and no longer to cortical lesion volume (eTable 5).

For significant associations between cortical lesions and baseline cognitive functioning, the association between cortical lesion volume and working memory (χ^2^ [2] = 13.6, *p* = 0.001) was moderated by MS disease stage, only showing significant effects of cortical lesion volumes in PMS (B [95% CI] = −0.48 [−0.75 to −0.22], *p* < 0.001).

#### Change in Cognition Explained by Baseline Cortical Lesion Count and Volume

Over 10 years, the log-transformed baseline cortical lesion count was significantly associated with cognitive decline (B [95% CI] = −0.18 [−0.32 to −0.04]), specifically in attention (B [95% CI] = −0.22[−0.40 to −0.03]) and verbal fluency (B [95% CI] = −0.23 [−0.37 to −0.09]; eTable 6). Baseline cortical lesion volume was associated with decline in average cognitive functioning (B [95% CI] = −0.08 [0.16 to −0.01]), specifically in attention (B [95% CI] = −0.11[−0.21 to −0.01]) and verbal fluency (B [95% CI] = −0.11[−0.18 to −0.03]). Including WM lesion volume to the linear mixed models did not significantly improve the model fits, indicating that WM lesion volume does not explain additional variance in cognitive functioning over 10 years.

Subsequently, an interaction term between baseline cortical lesion count/volume and MS disease stage was added as a fixed factor to the linear mixed model. None of the models provided a significantly improved model fit to the data, indicating the relationship between cortical lesions and cognitive change is consistent across the different MS disease stages.

#### Change in Cognition Explained by Change in Cortical Lesion Count

Over 10 years, increases in log-transformed cortical lesion counts were significantly associated with change in cognitive functioning (B [95% CI] = −0.21 [−0.40 to −0.03]), in particular verbal fluency (B [95% CI] = −0.33 [−0.51 to −0.15]; eTable 7). Differentiating between cortical lesion count increases from baseline to year 5 and from year 5 to year 10 did not significantly improve the model fit, reflecting no differential effects of early vs late cortical lesion count changes on cognitive trajectories. Including baseline cortical lesion counts as an additional covariate did not result in a significantly improved model fit either.

Then, an interaction term between change in cortical lesion count and MS disease stage was added as a fixed factor to the linear mixed models. None of the models yielded a significantly improved model fit. Hence, MS disease stage was not an effect modifier in the found relationships between change in cortical lesion count and cognitive decline during follow-up.

## Discussion

In this longitudinal study, we studied the evolution of function-specific cognitive functioning over 10 years in people with MS, focusing on the influence of cortical lesion load and formation on these trajectories and exploring whether the impact of cortical lesions on cognition differed across disease stages. Annualized decline varied between different cognitive functions, with greatest decline rates seen in verbal memory, followed by attention. Lowest decline rates were seen in those functions most severely affected already at baseline, that is, IPS and working memory. Cortical lesion counts increased over time, especially in those patients with higher cortical lesion load at baseline, with greater increases seen in the second 5-year period compared with the first 5 years. In the entire group, baseline cortical lesion load could explain baseline cognitive functioning of attention, EF, and verbal and working memory while IPS was more associated with WM lesion volume. While baseline cortical lesion load explained cognitive decline specifically in attention and verbal fluency, cortical lesion formation could only explain decline in verbal fluency over 10 years.

At baseline, people with MS exhibited worst functioning in IPS and working memory, and less so in verbal fluency and visuospatial memory, in line with recent literature.^1^ Like all MS symptoms, prevalence of cognitive impairment is highly heterogeneous between patients, but also across functions.^1^ Cognitive functioning was worse in progressive disease compared with early disease, especially in IPS, but this difference diminished over time. In other words, dysfunction of IPS was maximal near onset and stopped declining thereafter. Over 10 years, we observed a significant decline in attention, verbal memory, and EF, particularly in late relapsing-remitting and progressive disease phenotypes. Previous work studying cognitive profiles in MS introduced the potential of staging and stratifying cognition ordered from relatively preserved to impaired.^5,33-35^ This raised the concept of a potential order of events, leading to specific cognitive impairments,^33,34^ starting with IPS, followed by impairment in learning, working memory/attention, and eventually EF.^35^ This concept of a potential temporal order of impairment is supported by our findings, where we see impairment in IPS and working memory at baseline while decline over time is observed in functions relatively preserved at baseline. Remarkably, function-specific decline rates varied over time and across the different MS disease stages. This argues for nonlinear trends of cognitive changes and individual trajectories depending on different patterns of damage. In the light of individual trajectories, factors such as depression and fatigue should also be considered as relevant covariates when evaluating trends of cognitive changes, given their potential yet complex interrelationship in MS.^5,36^ More longitudinal studies are needed to establish the exact (annual) trends over time and to correlate these with other quantitative measures of damage and patient characteristics.

Most of the patients in our cohort formed new cortical lesions during follow-up. Cortical lesions significantly increased over time in all disease stages, with greater increases observed in progressive disease, which is in line with previous literature.^2,6,8^ Annualized increases in the cortical lesion count observed in our study (+0.4/year) are, as expected, lower compared with annualized increases seen at 7T field strength. Still, increases are somewhat similar to the rates seen in leukocortical lesions (+0.7/year)—the subtype most identified at our field strength^37^—compared with intracortical lesions (+1.1/year).^6^ People with MS with higher cortical lesion load at baseline had worse functioning in most cognitive functions at baseline. Considering the cognitive change over time, the impact of cortical lesions seems to shift toward the cognitive functions that (not yet) show decline over time. Specifically, higher baseline cortical lesion loads were related to change in attention and verbal fluency functioning over time, with a trend toward an association with verbal memory functioning. Increases in the cortical lesion count over time seemed only relevant for explaining changes in verbal fluency functioning. Previous work also found such a dynamic impact, as baseline cortical lesions formed early in the disease were associated with overall disability progression, whereas formation of new cortical lesions, except for the subpial subtype, was not associated with disability progression.^10^ In early MS (<5 years of disease duration), increases in cortical lesion volume over 2 years were associated with performance in attention, IPS, verbal memory, and verbal fluency in another study.^7^ The impact in later disease, however, has not been properly examined. As such, we specifically examined the relationships between cortical lesions and progression of decline in individual functions in a large and diverse MS sample. Nonetheless, longer term studies are needed to confirm the observed effects of cortical pathology on function-specific decline, at the time of lesion formation and during conversion toward progressive disease.

While higher cortical lesion formation and worse cognitive functioning were most evident in progressive MS, the associations between cortical lesion load and cognitive decline over time were not moderated by MS disease stage. Our findings suggest that cortical lesions have a different impact on function-specific cognitive functioning, potentially depending on this concept of an “order of impairments” and less depending on the clinical disease stage of an individual with MS. The differential impact might be attributed to varying susceptibility of cognitive functions to focal damage. It has been hypothesized that cognitive decline arises after reaching a point where the brain can no longer compensate for the accumulated damage.^38^ This threshold likely differs across cognitive functions. For instance, IPS may be more sensitive to WM disconnection compared with other cognitive functions,^39-41^ as highlighted by our findings. Cortically oriented cognitive functions are believed to depend more on cortical integrity, requiring more extensive microstructural cortical damage.^42^ The spatial patterns of damage that underlie the sequence of function-specific decline, however, remain unclear. Cortical involvement, encompassing not only cortical lesions but also cortical atrophy, consistently explains neurologic and cognitive impairments.^14,43^ As such, it would be worth exploring the interdependent contributions of regional cortical atrophy and cortical lesion development, providing a more comprehensive picture of cortical pathologies relevant for cognition.

This longitudinal study is not without limitations. It should be noted that there seemed to be a selective dropout of individuals already showing worse cognitive functioning at baseline compared with the ones who completed 10-year follow-up, which could affect possible differences between cross-sectional and longitudinal correlations. The smaller sample size at the 10-year follow-up requires careful interpretation, particularly in the context of the baseline and 5-year follow-up data. However, our mixed model analyses were performed only in those with 10-year follow-up. It would be of interest, however, to study the relationship between cortical pathology and cognitive decline in larger populations of progressive MS and in the earliest stages of the disease. In addition, the distinction between late RRMS and secondary PMS can be challenging, particularly given the overlap in clinical and radiologic features, underscoring the need for robust (longitudinal) biomarkers to better delineate these disease stages and reduce potential confounding in future studies. Likewise, distinguishing between early and late RRMS presents challenges in separating the effects of disease duration, as a function of time, from phenotype-specific impacts on cognitive decline and cortical lesion formation. The interaction between MS phenotypes and disease duration–related factors remains an intriguing avenue for future research. In addition, intracortical and especially subpial lesions remain difficult to visualize,^37,44^ so studying the impact of these lesions requires the development of additional imaging sequences.

In summary, our findings highlight that in our sample of people with MS, cognitive decline was worst in attention and verbal memory, while preexisting impairments were worst in other functions such as IPS, indicating a specific staging of impairments in individual functions. Cortical lesions developed throughout all stages of the disease. Worse baseline cognitive functioning was related to baseline cortical lesion load, whereas baseline cortical lesion load and longitudinal cortical lesion change were related to cognitive decline in functions not or less affected at baseline. These findings highlight the concept of an order of cognitive impairments, indicating that the spreading of impairments in individual cognitive functions is related to the severity of cortical pathology.

## Glossary

aiDIR: artificially generated double-inversion recovery
DIR: double-inversion recovery
EDSS: Expanded Disability Status Scale
EF: executive functioning
FLAIR: fluid-attenuated inversion recovery
GM: gray matter
HCs: healthy controls
IPS: information processing speed
IQR: interquartile range
MS: multiple sclerosis
PMS: progressive MS
RRMS: relapsing-remitting MS
WM: white matter
